# Evaluation of a real-time PCR assay for diagnosis of schistosomiasis japonica in the domestic goat

**DOI:** 10.1186/s13071-020-04420-8

**Published:** 2020-10-27

**Authors:** Qinghong Guo, Cheng Chen, Keke Zhou, Yugang Li, Laibao Tong, Yongcheng Yue, Kerou Zhou, Jinming Liu, Zhiqiang Fu, Jiaojiao Lin, Jiaxi Zhao, Pengxiang Sun, Yang Hong

**Affiliations:** 1 National Reference Laboratory of Animal Schistosomiasis, Key Laboratory of Animal Parasitology of Ministry of Agriculture, Shanghai Veterinary Research Institute, Chinese Academy of Agricultural Sciences, Shanghai, 200241 P. R. China; 2 Huancui Development Center for Animal Husbandry, Weihai, 264200 P. R. China; 3 Wangjiang County Center for Animal Disease Control and Prevention, Anqing, 246000 P. R. China; 4 Center for Disease Control and Prevention of Huancui, Weihai, 264200 P. R. China

**Keywords:** *Schistosoma japonicum*, Diagnosis, Real-time PCR, ELISA, Goats

## Abstract

**Background:**

Schistosomiasis japonica is an infectious disease caused by *Schistosoma japonicum* that seriously endangers human health. Domestic animals have important roles in disease transmission and goats are considered a primary reservoir host and source of infection. The prevalence and intensity of schistosomiasis infections have significantly decreased in China, and a more sensitive, specific detection method is urgently needed. The aim of this study was to develop a real-time PCR assay for accurate detection of *S. japonicum* infection in goats.

**Methods:**

A real-time PCR method for detecting schistosomiasis japonica in goats was developed by amplification of a specific *S. japonicum* DNA fragment, and validated using a total of 94 negative and 159 positive plasma and serum samples collected in our previous study of *S. japonicum* infection. Both plasma and serum samples were evaluated by real-time PCR and enzyme-linked immunosorbent assay (ELISA). In addition, 120 goat plasma samples from an *S. japonicum-*endemic area (Wangjiang) and 33 from a non-endemic region (Weihai) were collected and evaluated using our method.

**Results:**

The sensitivity and specificity of the real-time PCR for detecting infected samples were 98.74% (157/159, 95% CI: 95.53–99.85%) and 100% (94/94, 95% CI: 96.15–100%), respectively. For the ELISA, sensitivity and specificity were 98.11% (156/159, 95% CI: 94.59–99.61%) and 90.43% (85/94, 95% CI: 82.60–95.53%), respectively. Further, we found positivity rates for *S. japonicum* infection in Wangjiang and Weihai of 8.33% (10/120, 95% CI: 4.07–14.79%) and 0% (0/33, 95% CI: 0–10.58%), respectively.

**Conclusions:**

The results of this study indicate that our real-time PCR method exhibits higher sensitivity and specificity than ELISA and is a useful method for detection of *S. japonicum* infection in goats.
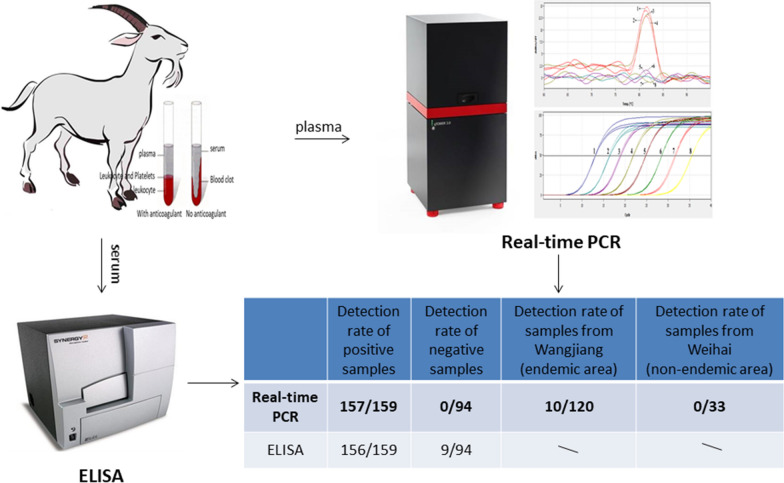

## Background

Schistosomiasis is a significant infectious disease caused by trematode worms of the genus *Schistosoma*, which is a global public health concern. Worldwide, schistosomiasis is the second most devastating parasitic disease after malaria, and it is mainly distributed in Asia, South America and Africa [1, 2]. The three major schistosome species that cause infection in humans are *Schistosoma japonicum*, *Schistosoma mansoni* and *Schistosoma haematobium*, among which, *S. japonicum* is mainly distributed in southeast Asian (China, The Philippines, etc.). According to previous studies, more than a million humans are infected with *S. japonicum*. Therefore, the disease remains a potential public health issue, despite considerable success in blocking transmission over the past decades [3–5].

Eradicating sources of infection is a vital strategy to eliminate schistosomiasis japonica [6]. Domestic animals are the primary source of infection and play an important role in disease transmission. In addition to bovines, goats are recognized as another major reservoir host in China [7]. Given the significantly decreasing prevalence and intensity of infection in China, development of a diagnostic method with high sensitivity and specificity for testing domestic animals is critical for effective control of schistosomiasis japonica [8].

Pathogenic and immunological diagnosis methods are used commonly for detection of *S. japonicum* infection. Pathogenic diagnosis primarily involves the Kato-Katz technique and miracidium hatching test (MHT), which have been regarded as the ‘gold standard’ for diagnosis of schistosomiasis [9, 10]. These approaches are used widely because of their low cost and portability; however, their disadvantages include low sensitivity in areas with low-intensity infections and the fact that they are relatively time-consuming [11]. Immunological diagnostic methods include enzyme-linked immunosorbent assay (ELISA) and the indirect hemagglutination (IHA) test, in addition to other methods based on the antibody and antigen reaction [12, 13]. These approaches have higher sensitivity and are simple to apply; however, they have higher cross-reactivity and false positive rates [14]. Given the limitations of pathogenic and immunological diagnosis methods, a more sensitive and specific detection technique is required for schistosomiasis diagnosis [15].

With the development of molecular diagnostic technology, nucleic acid detection of *S. japonicum* infection has become a research hotspot in recent years. Many studies have demonstrated that nucleic acid detection of *S. japonicum* infection has higher sensitivity and specificity, and less cross-reactivity than traditional methods [7, 16–18]. Molecular diagnostic techniques used include general PCR, nested-PCR, real-time PCR, loop-mediated isothermal amplification (LAMP), and recombinant enzyme polymerase amplification (RPA), among others [19]. In many previous studies, highly repetitive schistosome gene sequences were used as targets for nucleic acid detection of schistosomiasis. These sequences were mainly cell-free circulating schistosome DNA, derived from the dead worms during migration and the epidermal tissue cells, during their growth process [20–22]. In this study, a real-time PCR method for detecting *S. japonicum* DNA in goat plasma was developed and used to evaluate *S. japonicum* infection in goats from endemic and non-endemic regions.

## Methods

### Worm and egg collection

Adult *S. japonicum* worms (42 days post-infection) were collected from the portal vein systems of five BALB/c mice artificially infected with *S. japonicum* cercariae [23]. Male and female *S. japonicum* worms were manually separated and *S. japonicum* eggs collected from infected BALB/c mouse livers [24]. All *S. japonicum* worms and eggs were stored in RNAlater (Sigma-Aldrich, St. Louise, USA) at − 20 °C until use.

### Collection of plasma and serum samples

A total of 159 positive plasma and serum samples were simultaneously collected from artificially infected goats. The goat was restrained by a special restraint device, which could make it supine lying, preventing the animal from struggling. The abdominal wool was shaved off and an area of skin of approximately 10 × 10 cm was exposed. Then, goats were percutaneously infected with 300 cercariae for 20 min after counting the cercariae under a microscope. In addition, a total of 94 plasma and serum samples were collected from 94 goats without schistosome infection. Plasma and serum samples were collected respectively in EDTA-K2 vacuum blood collection tubes and a vacuum blood collection tubes with no additive. The blood was collected from jugular vein of each goat, and the supernatant was separated after centrifugation at 3000× *rpm* for 10 min at 25 °C. Plasma samples from 120 goats which grazed freely on marshlands were collected in Wangjiang county, which is a region endemic for schistosomiasis japonica in Anhui Province, China. Further, plasma samples from 33 goats which grazed freely on farms were collected in Weihai city, which is a non-endemic area for schistosomiasis japonica in Shandong Province, China. All plasma and serum samples were stored at − 20 °C until use. In addition, 32 positive plasma samples and their corresponding serum samples, as well as 10 positive plasma samples, were selected randomly for storage and use in later experiments.

### Plasma storage

The 10 positive plasma samples were randomly selected, divided into 4 aliquots of 6 ml each, and stored at 4 °C, − 20 °C, − 40 °C and − 80 °C, respectively. Samples were analyzed after 14 days, 28 days, 42 days, 3 months, 6 months, and 1 year of storage.

### Extraction of genomic and circulating DNA

Genomic DNA was extracted from the tissues of an uninfected goat, and from male and female *S. japonicum* worms and eggs using a TIANamp Genomic DNA Kit (Tiangen Biotech, Beijng, China), according to the manufacturer’s instructions. Genomic DNA samples from *Haemonchus contortus*, *Fasciola gigantica*, *Toxoplasma gondii*, *Sarcocystis* sp., *Trichinella spiralis*, *Paramphistomum*, *Babesia*, and *Spirometra* were provided by our institute and stored at − 20 °C.

DNA was extracted from goat plasma/serum samples using a Magnetic Serum/Plasma Circulating DNA Maxi Kit (Tiangen Biotech), according to the manufacturer’s instructions. Extracted DNA samples were stored at − 20 °C.

### Establishment and optimization of the real-time PCR detection system

**Fig. 1:**
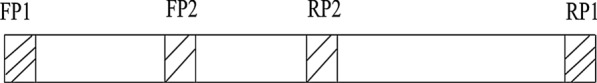
Relationship between two pairs of primers (FP1/RP1 and FP2/RP2)

PCR primers were designed according to a DNA fragment containing *S. japonicum* target sequence (GenBank: FN356222.1), which was verified through bioinformatics analysis and some screening experiments in our laboratory: forward primer 1 (FP1: 5ʹ-GCA GCG GCT TTA GGC AAC AC-3ʹ) and reverse primer 1 (RP1: 5ʹ-TCA AAC TAA TCC CTC TAT GGT TAT CAC AAG-3ʹ) (Fig. 1). PCR was performed using LA *Taq* DNA polymerase (Takara, Kyoto, Japan). The total reaction volume was 50 μl and included 5 μl 10× LA PCR Buffer II (Mg^2+^ plus), 8 μl dNTP mixture, 0.5 μl TaKaRa LA *Taq*, 1 μl each primer (FP1 and RP1), 32 μl ddH_2_O, and 2.5 μl DNA template. The reaction procedure comprised an initial denaturing step at 94 °C for 30 s, followed by 40 cycles at 94 °C for 15 s, 58 °C for 34 s, and 72 °C for 10 s.

PCR products were purified using a Universal DNA Purification Kit (Tiangen Biotech), according to the manufacturer’s instructions. The purified products were then subcloned into the pMD-19T vector overnight at 4 °C and recombinant plasmids transferred into *Escherichia coli* DH5α [25]. Recombinant plasmids were extracted using a Mini Plasmid Kit (Tiangen Biotech) and confirmed by DNA sequencing. Plasmid concentration was detected using an ultra-micro nucleic acid protein instrument (Thermo Fisher Scientific, Waltham, USA), plasmids were diluted to 2.6 ng/μl, and a 10-fold gradient dilution, up to 10^−9^, generated using sterile double distilled water for use in real-time PCR detection.

Real-time PCR primers were designed according to a specific DNA fragment used as target sequence (GenBank: FN356222.1), which was identified in our previous study (our unpublished data). The forward primer 2 (FP2: 5ʹ-CCG AAC ACT TCA AGG AAC AGT TTA G-3ʹ) and the reverse primer 2 (RP2: 5ʹ-CTT CCT CGT TTC AGG TTA GAT ATA GC-3ʹ) were used (Fig. 1). The total reaction system volume was 20 μl, including 10 μl 2× ChamQ Universal SYBR qPCR Master Mix (Vazyme Biotech, Nanjing, China), 0.4 μl (10 μmol/l) of each primer (FP2 and RP2), 5.2 μl ddH_2_O, and 4 μl (~20 ng) DNA template. The reaction procedure comprised an initial denaturing step at 94 °C for 30 s, followed by 40 cycles at 94 °C for 15 s, 58 °C for 34 s, and 72 °C for 10 s, and a melting curve step, comprising denaturation at 95 °C for 15 s, annealing at 60 °C for 15 s, and extension at 72 °C for 15 s using real-time PCR.

Optimization of annealing temperature was conducted using the *S. japonicum* target gene plasmid as a template, with annealing temperatures of 57 °C, 58 °C and 59 °C. To optimize plasma volume, 400, 500, 600 and 750 μl plasma from the same 10 positive goat samples were used. Furthermore, the amount of template DNA was optimized by extracting 10 nucleic acid samples from 600 μl plasma from infection-positive goats, using an annealing temperature of 58 °C and either 2 μl (approximately 10 ng) or 4 μl (approximately 20 ng) template.

### Detection of schistosomiasis japonica by real-time PCR

The plasmid containing the target sequence was used as a positive control. DNA from uninfected negative goat plasma was used as a negative control, and sterile double distilled water was used as a blank control in each assay. In each PCR detection, the positive control, the negative control and the blank control were included. All samples, positive control, negative control and blank control were detected in duplicate. Assays were performed using a qTOWER 3G instrument (Analytik Jena AG, Jena, Germany), and results determined using melting curve analysis. Samples were judged positive when both duplicate reactions were positive; suspicious when one reaction was positive, and sample nucleic acid re-extracted for a second detection, following which samples were judged as positive if one reaction was positive; and a sample was considered negative when two reactions were negative.

### Detection of schistosomiasis japonica by ELISA

*Schistosoma japonicum* soluble egg antigens (SEA) were prepared according to previously described methods [26]. SEA were the detection antigen and all serum samples were tested by ELISA. First, SEA were diluted appropriately with coating solution, used to coat 96-well microtiter plates (100 μg per well; Corning, Corning, USA) overnight at 4 °C, then washed three times with 0.05% Tween 20 in PBS (PBST). Next, plates were blocked with 1% BSA (100 μl/well), incubated for 1 h at 37 °C, then washed three times. Serum samples were diluted 1:100 with PBS, added (100 μl/well), incubated for 2 h at 37 °C, and washed three times. HRP-labeled donkey anti-goat IgG (H+L) (Beyotime, Shanghai, China) diluted 1:3000 in PBST was added (100 μl/well), incubated for 1 h at 37 °C, and then washed three times. Finally, 100 μl/well soluble TMB substrate solution (Tiangen Biotech) was added, plates incubated in the dark for 5–10 min, and reactions stopped by adding 2 M sulfuric acid (50 μl/well). Optical density (OD) was measured at 450 nm on a microplate reader (BioTek, Vermont, USA). In each ELISA detection, a positive serum control and a negative serum control were included. All samples, positive serum control and negative serum control were detected in triplicate. The mean absorbance value of the reference negative serum multiplied by 2.1 was set as the cutoff value. A sample was judged positive when its mean absorbance value was higher than the cutoff value.

### Statistical analysis

Sensitivity and specificity were calculated as follows: sensitivity = no. of true positives/(no. of true positives + false negatives); specificity = no. of true negatives/(no. of true negatives + false positives). Stata/SE 12.0 was used to calculate 95% confidence intervals (CI) for the sensitivity and specificity of each detection. The mean and standard deviation (SD) were calculated. Comparisons among methods of analysis were conducted using a Chi-square test in SPSS Statistics (version 20). A value of *P *< 0.05 was considered statistically significant.

## Results

### Establishment of a real-time PCR detection system to detect *S. japonicum*

**Fig. 2:**
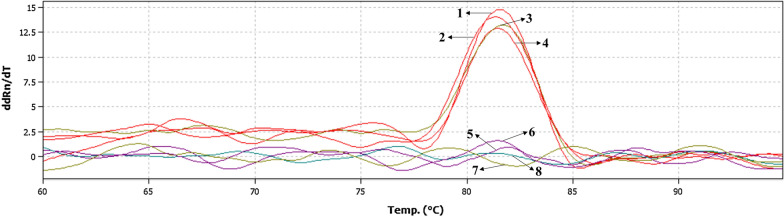
Real-time PCR melting curves. *Key*: 1, positive control; 2–4, positive samples; 5–6, negative samples; 7, negative control; 8, blank control

The real-time PCR results were determined using melting curve analysis (Fig. 2). The results showed that the positive detection rates were 100% (10/10) at annealing temperatures of 57 °C and 58 °C; however, the melting curve for the reaction had more than one peak at 57 °C. At an annealing temperature of 59 °C, the detection positive rate was 90% (9/10) and the melting curve had single peak. Therefore, we concluded that 58 °C was the optimum annealing temperature.

The positive detection rates using 400, 500, 600 and 750 μl goat plasma samples were 60% (6/10), 80% (8/10), 100% (10/10) and 100% (10/10) by real-time PCR; hence, the optimal plasma volume was determined as 600 μl. Real-time PCR was performed using 2 μl (*c.*10 ng) and 4 μl (*c.*20 ng) template, and the detection positive rates were 80% (8/10) and 100% (10/10), respectively; hence, the final optimal template amount was determined as 4 μl.

### Detection using plasma and serum samples

Nucleic acids samples were extracted from 32 positive goat plasma and corresponding serum samples and analyzed by real-time PCR detection using the same conditions. The results showed that all 32 plasma samples were positive (32/32, 95% CI: 89.11–100%) and 27 serum samples were positive (84.38%, 27/32, 95% CI: 67.21–94.72%). These data indicate that plasma samples were more suitable for detection than serum samples.

### Effects of plasma storage

Next, real-time PCR was performed using samples stored in different environments and for various lengths of time. After storage of plasma samples for 14 days at 4 °C, − 20 °C, − 40 °C and − 80 °C, detection rates were 100% (10/10). Nine samples were positive (90%, 9/10) following storage at 4 °C after 28 days, while detection rates of samples stored at the three other temperatures were 100% (10/10). Three samples were positive (30%, 3/10) after storage at 4 °C for 42 days, while detection rates for samples stored at the other three temperatures were 100% (10/10); the detection rates for all samples stored at − 20 °C, − 40 °C, and − 80 °C were 100% (10/10) after 3 months, 6 months, and 1 year, respectively (Table 1).

**Table 1:** Results of real-time PCR analysis of plasma following storage for various time periods and at different temperatures

Storage time	Temperature			
	4 °C	− 20 °C	− 40 °C	− 80 °C
14 days	100%	100%	100%	100%
28 days	90%	100%	100%	100%
42 days	30%	100%	100%	100%
3 months	na	100%	100%	100%
6 months	na	100%	100%	100%
12 months	na	100%	100%	100%

*Abbreviation*: na, no detection

### Sensitivity and specificity of real-time PCR and ELISA

**Fig. 3:**
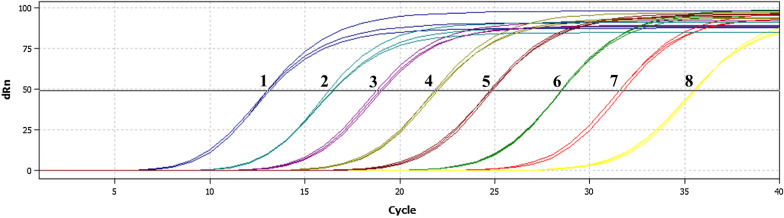
Real-time PCR amplification curves for the positive control plasmid at different dilutions. *Key*: 1–8, plasmid diluted at 1 (1 ng/μl); 10^−1^ (100 pg/μl); 10^−2^ (10 pg/μl); 10^−3^ (1 pg/μl); 10^−4^ (100 fg/μl); 10^−5^ (10 fg/μl); 10^−6^ (1 fg/μl); and 10^−7^ (0.1 fg/μl), respectively

The results of real-time PCR showed that there was no target gene amplification from goat genomic DNA, while the assay could be used to detect schistosomiasis japonica in goats. Sample concentration was calculated using a standard curve based on a 10-fold gradient dilution of the positive control plasmid. The amplification curve (Fig. 3) indicated that the lowest detection level was 10^−7^ dilution, with a corresponding copy number of 74.9. The copy numbers of the target gene in one egg, a single male, and a single female of *S. japonicum* were (8.73 ± 0.03) × 10^6^, (8.61 ± 0.03) × 10^9^, and (5.51 ± 0.03) × 10^9^, respectively.

The sensitivity and specificity of real-time PCR were 98.74% (157/159, 95% CI: 95.53–99.85%) and 100% (94/94, 95% CI: 96.15–100%), respectively. Meanwhile, the sensitivity and specificity of ELISA were 98.11% (156/159, 95% CI: 94.59–99.61%) and 90.43% (85/94, 95% CI: 82.60–95.53%), respectively. There was no significant difference in sensitivity between real-time PCR and ELISA for detecting goat schistosomiasis japonica (*P* = 0.652); however, there was a significant difference in the specificity of the two methods in goat samples (*P* = 0.002).

### Cross-reactivity of real-time PCR

PCR assays using genomic DNA of eight parasite species (*H. contortus*, *F. gigantica*, *T. gondii*, *Sarcocystis* sp., *T. spiralis*, *Paramphistomum*, *Babesia* and *Spirometra*) as template were negative, with no amplification of the target gene detected. Thus, our real-time PCR method exhibited no cross-reactivity with these organisms.

### Real-time PCR detection using samples from goats from endemic and non-endemic areas

Our real-time PCR method was used to analyze 120 plasma samples from goats from an endemic area (Wangjiang) and 33 from goats from a non-endemic area (Weihai). The results showed that the positive rate of samples from Wangjiang was 8.33% (10/120, 95% CI: 4.07–14.79%), while that in Weihai was 0% (0/33, 95% CI: 0–10.58%).

## Discussion

More than 40 mammalian species can be reservoir hosts of *S. japonicum*, and goats are an important source of human infection. Due to large-scale treatment with praziquantel, safety education, and extensive comprehensive control strategies, the prevalence and infection intensity of schistosomiasis japonica have been reduced successfully and maintained at a very low level [27, 28]; however, the disease continues to spread, although at a very low-intensity of infection, and eggs in the feces of goats are one of the main sources of transmission [29].

Currently, no *S. japonicum* diagnosis is available with easy field access and high sensitivity and specificity in low-intensity infection areas [30]. Thus, schistosomiasis diagnosis remains challenging in areas of low infection intensity [31, 32]. The real-time PCR method has numerous advantages for diagnosis, including its high sensitivity, high specificity, low cross-reactivity with other parasites, and the fact that the results can be evaluated directly, by the melting curve. *Schistosoma japonicum* can infect many mammals; however, not all have corresponding commercial secondary antibodies. Thus, the use of immunological methods to diagnose schistosomiasis japonica is limited in different animals. In contrast, the real-time PCR method detects *S. japonicum* nucleic acid, providing the potential advantage for schistosomiasis diagnosis in a range of animals.

In this study, we developed a real-time PCR diagnostic method to detect schistosomiasis japonica in goat plasma samples. The real-time PCR target gene was a specific DNA fragment, previously screened in our laboratory (data not shown). This gene could be detected in schistosomiasis japonica-positive plasma, and there was no cross-reaction with goat genomic DNA. Therefore, we determined that it was suitable for use in detecting schistosomiasis japonica in goats.

In previous studies, host serum was generally used for extraction of nucleic acids and PCR detection [33–35]. In the present study, plasma and serum samples from schistosomiasis-positive goats were analyzed. The detection was higher in goat plasma than that in serum. This may be because cell-free DNA is more stable in plasma than serum, and DNase activity is very high in serum, leading to rapid degradation of cell-free DNA. Further, the EDTA used in the preparation of plasma is a DNase inhibitor, and can inhibit cell-free DNA degradation in plasma. Moreover, plasma contains more fibrinogen and clotting factors than serum, and it is possible that serum can drag down a proportion of cell-free DNA during the clotting process, leading to a loss of cell-free DNA in serum compared with plasma [36, 37].

The influences of storage temperature and time on plasma samples were also evaluated in this study. Positive plasma samples were stable at least for one year when stored at − 20 °C, − 40 °C and − 80 °C. The detection rate was 100% within two weeks when samples were stored at 4 °C; however, after four weeks at 4 °C, detection rates declined. Therefore, plasma samples should not be stored for over two weeks at 4 °C, and can be used for at least for one year if stored at − 20 °C, − 40 °C, or − 80 °C.

The lower limit of detection for the real-time PCR method was 0.26 fg target gene, with a corresponding copy number of 74.9. Moreover, in single male and female mean *S. japonicum* genome copy numbers were (8.61 ± 0.03) × 10^9^ and (5.51 ± 0.03) × 10^9^, respectively, while a single egg contained only (8.73 ± 0.03) × 10^6^ copies. In previous study, we reported a nested-PCR method that can detect target DNA from a single egg; however, it required two-rounds of reactions, which could increase the risk of contamination [7]. In contrast, the real-time PCR assay developed here only requires a single-round reaction and the results can be judged directly by melting curve analysis, which is a potentially significant advantage.

In this study, the real-time PCR exhibited slightly better performance than ELISA for detection of *S. japonicum*-positive samples. There was no significant difference in sensitivity between real-time PCR and ELISA for detection of schistosomiasis japonica-positive samples from goats (*P* = 0.652); however, there was a significant difference in specificity between the two methods (*P* = 0.002). Hence, real-time PCR may improve the specificity of schistosomiasis diagnosis in goats, with superior specificity compared with ELISA.

In addition, the real-time PCR method did not cross-react with *H. contortus*, *F. gigantica*, *T. gondii*, *Sarcocystis* sp., *T. spiralis*, *Paramphistomum*, *Babesia* and *Spirometra*. Nevertheless, in some previous studies, ELISA methods cross-reacted with *H. contortus* in goats and *Paramphistomum* in water buffaloes [13, 38]. Thus, our real-time PCR method has the potential to improve the detection accuracy compared with that achieved using an ELISA approach.

Finally, this method was used to evaluate the goats from endemic and non-endemic regions. No samples from Weihai tested positive, while ten samples from Wangjiang did. In a previous study, a nested-PCR method was also used to evaluate the schistosomiasis japonica infection in goats from Wangjiang, and the positive rate was 16.67% [7]. Although real-time PCR may be a more sensitive method than nested-PCR, the positive rate using real-time PCR was slightly lower (8.33%) than that using nested-PCR. We speculate that this may be due to the considerable efforts made to control and prevent schistosomiasis japonica in goats in Wangjiang; however, our data demonstrate that schistosomiasis japonica infection in goats in endemic regions remains at a high level and warrants further attention. Plasma samples from goats from Weihai were all negative, confirming that the real-time PCR method has excellent specificity. In this study, DNA extracted from the plasma of goats was finally selected for detection after comparing the diagnostic effect of plasma and serum from goats. Meanwhile, to the best of our knowledge, this is the first time schistosomiasis japonica has been diagnosed in goats by real-time PCR. The sensitivity and specificity of this method was not worse than other PCR methods that have been used to diagnose schistosomiasis in human or other animals for detecting DNA from different samples. However, real-time PCR also has some shortcomings, such as reliance on expensive instruments and its higher cost, which would be resolved with continuous development.

## Conclusions

The real-time PCR method developed in this study has higher sensitivity and specificity than previously published approaches and has potential for application for detection of *S. japonicum* infection in domestic animals, particularly goats.

## Data Availability

Data supporting the conclusions of this article are included within the article.

## Abbreviations

ELISA: enzyme-linked immunosorbent assay
MHT: miracidium hatching test
IHA: indirect hemagglutination assay
LAMP: loop-mediated isothermal amplification
RPA: recombinant enzyme polymerase amplification
SEA: soluble egg antigen
